# PSA-NCAM positive neural progenitors stably expressing BDNF promote functional recovery in a mouse model of spinal cord injury

**DOI:** 10.1186/s13287-015-0268-x

**Published:** 2016-01-13

**Authors:** Jennifer Butenschön, Tina Zimmermann, Nikolai Schmarowski, Robert Nitsch, Barbara Fackelmeier, Kevin Friedemann, Konstantin Radyushkin, Jan Baumgart, Beat Lutz, Julia Leschik

**Affiliations:** Institute of Physiological Chemistry, University Medical Center, Johannes Gutenberg University, Duesbergweg 6, 55128 Mainz, Germany; Institute of Microscopic Anatomy and Neurobiology, University Medical Center, Johannes Gutenberg University, Langenbeckstrasse 1, 55131 Mainz, Germany; Mouse Behavior Outcome Unit, Focus Program Translational Neurosciences (FTN), Johannes Gutenberg University, Hanns-Dieter-Hüsch-Weg 19, 55128 Mainz, Germany; Translational Animal Research Center (TARC), University Medical Center, Johannes Gutenberg University, Hanns-Dieter-Hüsch-Weg 19, 55128 Mainz, Germany

**Keywords:** Spinal cord contusion injury, Brain-derived neurotrophic factor, Neural differentiation, Embryonic stem cells

## Abstract

**Background:**

Neural stem cells for the treatment of spinal cord injury (SCI) are of particular interest for future therapeutic use. However, until now, stem cell therapies are often limited due to the inhibitory environment following the injury. Therefore, in this study, we aimed at testing a combinatorial approach with BDNF (brain-derived neurotrophic factor) overexpressing early neural progenitors derived from mouse embryonic stem cells. BDNF is a neurotrophin, which both facilitates neural differentiation of stem cells and favors regeneration of damaged axons.

**Methods:**

Mouse embryonic stem cells, modified to stably express BDNF-GFP, were differentiated into PSA-NCAM positive progenitors, which were enriched, and SSEA1 depleted by a sequential procedure of magnetic-activated and fluorescence-activated cell sorting. Purified cells were injected into the lesion core seven days after contusion injury of the spinal cord in mice, and the Basso mouse scale (BMS) test to evaluate motor function was performed for 5 weeks after transplantation. To analyze axonal regeneration the anterograde tracer biotinylated dextran amine was injected into the sensorimotor cortex two weeks prior to tissue analysis. Cellular differentiation was analyzed by immunohistochemistry of spinal cord sections.

**Results:**

Motor function was significantly improved in animals obtaining transplanted BDNF-GFP-overexpressing cells as compared to GFP-expressing cells and vehicle controls. Stem cell differentiation in vivo revealed an increase of neuronal and oligodendrocytic lineage differentiation by BDNF as evaluated by immunohistochemistry of the neuronal marker MAP2 (microtubule associated protein 2) and the oligodendrocytic markers ASPA (aspartoacylase) and Olig2 (oligodendrocyte transcription factor 2). Furthermore, axonal tracing showed a significant increase of biotin dextran amine positive corticospinal tract fibers in BDNF-GFP-cell transplanted animals caudally to the lesion site.

**Conclusions:**

The combinatorial therapy approach by transplanting BDNF-overexpressing neural progenitors improved motor function in a mouse contusion model of SCI. Histologically, we observed enhanced neuronal and oligodendrocytic differentiation of progenitors as well as enhanced axonal regeneration.

**Electronic supplementary material:**

The online version of this article (doi:10.1186/s13287-015-0268-x) contains supplementary material, which is available to authorized users.

## Background

Spinal cord injury (SCI) is in most cases caused by contusion and compression of the spinal cord, leading to complete or incomplete loss of motoric and sensory functions. Besides the lack of neuronal regeneration of severed axons owing to inhibitory molecules, massive cell death occurs in particular of neurons and oligodendrocytes. Death of oligodendrocytes can be regarded as particularly harmful, because this can lead secondarily to demyelination of spared, intact axons, in cases of prevalent incomplete injury [1].

Cell therapy is regarded as one potential therapeutic approach for SCI. Diverse cell types have been tested already in transplantation studies in SCI animals, such as Schwann cells, olfactory ensheathing cells, mesenchymal stem cells, and embryonal stem cells [2]. However, none of these cell types led to a striking functional motoric improvement. Hereby, embryonic stem cells (ESCs) (differentiated into neural stem cells (NSCs)) were the most promising cell source, because they could at least lead to a moderate functional improvement [3–6]. The advantage of ESCs compared with other cell types, such as adult stem cells (e.g., mesenchymal stem cells) or other somatic cells (e.g., Schwann cells or olfactory ensheathing cells), is their high differentiation potential in diverse cell types and their better survival rate in vivo. However, the only moderate improved function could possibly be explained by the fact that not enough ESCs have differentiated into the required specialized cell type or that cell replacement was not sufficient to compensate for the limited regeneration potential of the spinal cord. Combinatorial approaches (e.g., coapplication of neurotrophins) might therefore be necessary for complete functional restoration. The neurotrophin brain-derived neurotrophic factor (BDNF) is a secreted growth-promoting (“trophic”) protein which is particularly important for neuronal survival and differentiation [7–10]. Furthermore, it is known that BDNF is important for oligodendroglial proliferation, differentiation, and myelination [11–13]. In addition, by using BDNF-secreting fibroblasts or by viral delivery, BDNF was shown to have a positive effect on axonal regeneration [14–17] and to enhance the integration of transplanted embryonal precursor cells [18].

In a previous study [19], we demonstrated that ESCs stably expressing BDNF–green fluorescent protein (GFP) display an enhanced neuronal differentiation in vitro. The current study addresses the use of these ESCs in a contusion mouse model of SCI. This approach involves a combination therapy using ESC-derived polysialylated neural cell adhesion molecule (PSA-NCAM)-positive neural progenitors, which first have the potential to differentiate into neurons and oligodendrocytes [20], and second guarantee a long-term exogenous supply of BDNF. Here, we report that BDNF-overexpressing transplanted progenitors differentiated in vivo to an increased extent into neurons and oligodendrocytes compared with control cells. Furthermore, the numbers of corticospinal tract (CST) fibers caudal to the lesion site were enhanced by transplantation of BDNF-overexpressing progenitors, indicative for axonal regeneration. As a result of histopathological improvements, recovery of motor function was obtained only in the group transplanted with BDNF-overexpressing progenitors. Tests for mechanical induced allodynia revealed that high expression of BDNF was not harmful in terms of pain threshold. Furthermore, we did not observe any tumor formation because we used a defined protocol of ESC differentiation (modified from [21]) and subsequent purifications to yield a pure population of PSA-NCAM-positive progenitors [22].

## Methods

### Differentiation of ESCs

C57BL/6 mouse ESCs were modified by knock-in technology into the Rosa26 locus to overexpress either BDNF-GFP or GFP as described previously [19]. ESCs were maintained on feeder cells (mouse embryonic fibroblasts) and cultured in the presence of leukemia inhibitory factor (LIF) under standard conditions [23]. For differentiation, cells were cultured for two passages on gelatin and then differentiated according to the protocol of [21] with minor modifications to yield PSA-NCAM-positive neural progenitors. Briefly, cells were dissociated and reaggregated to form embryoid bodies (EBs) for 6 days on nonadherent bacterial-grade dishes. EBs were then plated on adherent dishes and kept in ITSFn medium (insulin, transferrin, selenium, and fibronectin) in Dulbecco’s modified Eagle’s medium (DMEM)/F12 to select for Nestin-positive cells. After 8 days, cells were trypsinized and replated onto dishes coated with poly-ornithin/laminin (Sigma Aldrich, St. Louis, MO, USA) at a density of 1.5 × 10^5^ cells/cm^2^ in DMEM/F12/Glutamax supplemented with B27 (all from Invitrogen, Carlsbad, CA, USA) and 10 ng/ml basic fibroblast growth factor (bFGF; Sigma Aldrich). Cells were expanded for 6 days. Thereafter, cells were purified by the sequential use of magnetic-activated cell sorting (MACS; Miltenyi Biotec, Bergisch Gladbach, Germany) and fluorescence-activated cell sorting (FACS) technology.

### Purification of progenitors by MACS and FACS

#### MACS

MACS (Miltenyi Biotec) purification was performed in two steps. First, undifferentiated ESCs were labeled with anti-stage-specific embryonic antigen-1 (anti-SSEA-1) magnetic MicroBeads to remove undifferentiated ESCs with tumorigenic potential. After trypzination of cells, 20 μl anti-SSEA-1 MicroBeads/10^7^ cells were added and incubated for 15 minutes at 4 °C. After washing and resuspension in MACS buffer, labeled cells were applied to the LD Column and the flow through (unlabeled cells) was collected. Second, PSA-NCAM-positive progenitors were enriched by incubating the SSEA-1-negative cell population with 10 μl PSA-NCAM-allophycocyanin (APC)/10^7^ cells for 10 minutes at 4 °C. Then, 20 μl anti-APC magnetic MicroBeads/10^7^ cells were added. After 15 minutes of incubation at 4 °C, cells were washed and resuspended in MACS PB buffer, and then applied to the LS Column. PSA-NCAM-positive cells retained in the column and were collected by removal of the magnet from the column.

#### FACS

To further increase the purity of the MACS-enriched PSA-NCAM-positive cell population, PSA-NCAM-APC-labeled cells were incubated with r-phycoerythrin (PE)-conjugated SSEA-1 antibody (Stemgent, Cambridge, MA, USA) for 1 hour at 4 °C. Cells were washed with Dulbecco’s Phosphate-Buffered Saline (DPBS), resuspended in DPBS (7 × 10^6^ cells/ml for sorting), and filtered through a 30 μm preseparation filter. Stained cells were sorted on a fluorescence-activated cell sorter BD FACSAria™ III, using FACSDiva™ Software (BD Biosciences, Heidelberg, Germany). The sorting speed was adjusted to ensure sorting efficiency above 90 %. Forward scattering and side scattering excluded debris and dead cells. The following negative controls were used to identify the population of interest: unstained cells before MACS; stained cells with SSEA-1 antibody before MACS; and PSA-NCAM-APC only (cells after PSA-NCAM enrichment, no SSEA-1 stain). SSEA-1^–^/PSA-NCAM^+^ FACS sorted cells were plated onto poly-ornithin/laminin coated dishes at a density of 1.5 × 10^5^ cells/cm^2^.

### Flow cytometric analysis and immunocytochemistry

To determine the amount of PSA-NCAM and SSEA-1-positive cells in the expansion culture, cells were analyzed via flow cytometry and immunofluorescence. The following antibodies were used: primary mouse IgM monoclonal PSA-NCAM (1:100; Chemicon, Billerica, MA, USA), primary mouse IgM monoclonal SSEA-1 (1:50; Developmental Studies Hybridoma Bank, University of Iowa, Iowa City, IA, USA), or secondary antibody anti-mouse AlexaFluor546 IgM (1:1000; Invitrogen).

For flow cytometric analysis before MACS/FACS purification, cells were trypsinized and gently triturated. Cells were filtered through a 30 μm preseparation filter to obtain single cell suspensions. Then 1 × 10^6^ cells were fixed for 15 minutes with 4 % paraformaldehyde (PFA), washed, and incubated with the primary antibody for 1 hour. Subsequently, cells were incubated with the secondary antibody for 45 minutes. All washing and incubation steps were performed in 0.5 % bovine serum albumin/phosphate-buffered saline (PBS) at room temperature. Stained cells were analyzed on a flow cytometer (BD LSRFortessa™). PSA-NCAM and SSEA-1 positivity was determined according to negative controls where no primary antibody was used and by comparison with undifferentiated ESCs, which do not express PSA-NCAM but SSEA-1.

For immunofluorescence staining before and after MACS/FACS purification, cells were fixed in 4 % PFA for 15 minutes, rinsed with PBS, and incubated in 4 % goat serum in PBS for 15 minutes. Cells were then incubated with primary antibodies in 4 % goat serum overnight at 4 °C. After 3 × 5 minute PBS washes, cells were incubated with the secondary antibody for 1 hour at room temperature. After rinsing with PBS, 4′,6-diamidino-2-phenylindol (DAPI) was used for nuclear counterstaining and coverslips were mounted with Mowiol.

For immunofluorescence of 5-bromo-2′-deoxyuridine (BrdU)-treated cells before transplantation, cells were processed according to BrdU immunohistochemistry of spinal cord sections.

### BrdU treatment of cells and preparation of cells for transplantation

To ensure cell detection after transplantation, cells were labeled with BrdU (Sigma Aldrich). One day after FACS sorting, cells were incubated with 1 μg/ml BrdU for 48 hours. For transplantation, cells were trypsinized, washed with DPBS, and resuspended in Hank’s Balanced Salt Solution without calcium and magnesium (HBSS w/o Ca^2+^/Mg^2+^; Sigma Aldrich) at a concentration of 1 × 10^5^ cells/μl.

### Animals and contusion SCI

All surgical interventions and animal care were provided in accordance with the German Committee on Animal Health and Care of Rhineland-Palatinate (application number: G 14-1-012).

Thirteen-week-old male C57BL/6 mice were analgized (Rimadyl, Pfizer, New York City, NY, USA, 4 mg/kg;) and anesthetized with 3 % isoflurane, and the spinal cord was exposed at the T8 vertebral level via a laminectomy. With a stereotactic system and a blunt knife (2 mm long, 1 mm wide), a force of 30 × *g* for 1 minute was placed on the spinal cord to induce a severe contusion injury. Sham mice were not subjected to a contusion injury but to a laminectomy. The inner suture was performed with an atraumatic suture material. The skin suture was closed with a reflex wound clip system. Postsurgical care included at least 10 days of subcutaneous saline injection to maintain hydration and manual bladder expression once a day until spontaneous voiding returned.

### Transplantation

Seven days after surgery, mice were either treated with vehicle injection (HBSS w/o Ca^2+^/Mg^2+^) or received cell transplants directly into the lesion core. Mice were analgized and anaesthetized as described for contusion surgery. After disinfection of the back skin the suture was reopened. Then 1 μl HBSS w/o Ca^2+^/Mg^2+^ or 1 × 10^5^ cells/μl HBSS w/o Ca^2+^/Mg^2+^ were injected by self-made glass capillary with a tip 70–90 μm in diameter configured to a 10 μl Hamilton syringe and a small animal stereotaxic injection system (David Kopf Instruments, Tujunga, CA, USA). The cell suspension or vehicle solution was injected into the lesion core at the T8 level over a 5-minute period with an injection rate of 200 nl/minute. The syringe was maintained in place for an additional 5 minutes to prevent back-flux from the injection site. The surgery site was closed as already described.

### Anterograde tracing

Sixteen days prior to processing the animals for histological analysis, the nontoxic, axonal tracer biotinylated dextran amine (BDA) was injected into the sensorimotor cortex. After shaving and disinfection of the skin, the scalp was removed by cutting in a rostrocaudal direction.

Injection coordinates were 1.0 mm lateral to the midline at 0.5 mm anterior, 0.5 mm posterior, and 1.0 mm posterior to bregma at a depth of 0.5 mm from the cortical surface. Six small holes were drilled in the skull over the sensorimotor cortex. Then 0.2 μl tetramethylrhodamine and biotin-conjugated dextran amine (10,000 MW, lysine (mini ruby); Invitrogen) was injected per injection hole into the sensorimotor cortex with a 10 μl Hamilton syringe fitted with a pulled glass capillary. The skin suture was closed with a reflex wound clip system. For analysis of tracing, see Microscopic analysis of histology.

### Behavioral assays

#### Basso mouse scale

To assess motor function of the hindlimbs, the Basso mouse scale (BMS) was used [24]. All mice were pretrained and tested in a round open field (120 cm in diameter) preoperatively, 24 hours after SCI and at least weekly for up to 42 days post operation (DPO). Two independent raters, who were blinded to the experimental conditions, evaluated functional recovery using the BMS. Each mouse was observed separately for 4 minutes in each session and hindlimb movements were assessed with the scale ranging from 0 (no ankle movement) to 9 (complete functional recovery) points. The two scores for left and right hindpaws were averaged to obtain a single value per mouse, which represents the mobility of the mouse. Mice with a BMS score higher than 3 at 24 hours after injury were excluded from future evaluation (*n* = 2).

#### Von Frey filament test

Mechanical allodynia was tested using the von Frey filament test, which measures nociceptive responses in rodents after application of a non-noxious mechanical stimulus to the hindpaw. The frequency of paw withdrawal was measured by stimulating the plantar surface of the right and left hindpaws with filaments of different weight (filament weight range 0.6–8.0 g; Ugo Basile, Varese, Italy). The pain threshold was established using the up–down method as described previously [25]. The test was carried out in all mice 42 days after SCI. The mouse was placed in a clear plastic box with a metal mesh floor and was allowed to habituate to the experimental environment for 45 minutes before carrying out the test. Von Frey filaments were then applied from underneath the metal mesh floor to stimulate the plantar surface of the paw.

The test started by using the von Frey filament of 1.4 g corresponding to the middle mechanical force applied among the selected range of filaments (0.6–8.0 g). A response (paw withdrawal) in at least three out of five stimulations was considered positive. If a positive response upon application of the 1.4 g filament force was found, a lower weight filament (<1.4 g) was applied to determine the response of the animal to the minimal force applied (pain threshold). In the case of a negative response upon application of the 1.4 g weight filament, filaments of increasing force (>1.4 g) were applied until the threshold was determined.

Only animals in which a pain threshold in at least one hindpaw could be measured were plotted, resulting in a lower number than in the BMS. The pain threshold was considered not measurable when a filament force above 8 g was required to elicit a response.

### Immunohistochemistry

Immediately after transcardial perfusion with 4 % PFA (performed at DPO43), the columna vertebralis was excised from the mouse body and fixated in 4 % PFA overnight. On the next day, the spinal cord was excised from the columna vertebralis by cutting off the spines and breaking off the bones. After PFA fixation overnight, PFA was exchanged with 15 % sucrose followed by 30 % sucrose (each for 1 day) until usage. Then 20 μm sagittal sections were cryosectioned and directly mounted on (SuperFrostPlus, Menzel, Braunschweig, Germany) slides.

For immunohistochemistry, sections were fixed in 4 % PFA for 15 minutes, rinsed for 5 minutes in 0.2 % PBS-TX, and incubated with 4 % goat serum for 15 minutes. Sections were then incubated with primary antibodies overnight at 4 °C in 4 % goat serum. The following primary antibodies were used: rabbit polyclonal anti-microtubule-associated protein 2 (anti-MAP2; 1:200) and monoclonal anti-oligodendrocyte transcription factor 2 (anti-Olig2, 1:100; both from Chemicon, Billerica, MA, USA), rabbit polyclonal anti-glial fibrillary acidic protein (anti-GFAP, 1:1000; Abcam, Cambridge, UK), and rabbit polyclonal anti-aspartoacylase (anti-ASPA; 1:1000) and rabbit polyclonal anti-GFP (1:1000; kind gifts from Matthias Klugmann, Sydney, Australia). After 3 × 5 minute 0.2 % PBS-TX washes, the sections were incubated with the secondary antibody (goat anti-mouse or anti-rabbit AlexaFluor546, 1:1000; Invitrogen) for 1 hour at room temperature. Then 3 × 5 minute 0.2 % PBS-TX washes were performed. To double-stain with an anti-BrdU antibody, slides (stained for all antibodies except BrdU) were fixed again for 15 minutes in 4 % PFA to stabilize antigen–antibody complexes. Sections were then incubated in 1 N HCl for 1 hour at 37 °C to denature DNA, followed by 3 × 10 minute PBS washes. Sections were further incubated in blocking solution containing 1 % donkey serum for 90 minutes. After blocking, the incubation with the rat monoclonal anti-BrdU antibody (1:100; Abcam) was performed overnight at 4 °C. The second day, slides were rinsed for 3 × 10 minutes with PBS before the sections were incubated with the secondary antibody (goat anti-rat AlexaFluor647, 1:1000; Invitrogen) for 90 minutes, and then washed for 2 × 10 minutes with PBS. The background was reduced by counterstaining with 0.05 % Sudan black for 5 minutes. The sections were then washed with 70 % ethanol and dH_2_O. Slides were mounted with Mowiol.

### Microscopic analysis of histology

Slides were observed under a Leica DM5500 (Leica Camera, Wetzlar, Germany) fluorescence microscope or a Zeiss Axiovert LSM 710 (Carl Zeiss, Oberkochen, Germany) laser scanning confocal microscope. For laser scanning confocal microscopy, z-stacks with optical sections of 2 μm of the lesion core and adjacent regions were recorded.

Lesion volume and cell survival were assessed based on the Cavalieri principle of stereology. To determine the lesion volume, every 10th section of the spinal cord was stained for the astrocytic marker GFAP and digital photomicrographs were taken. The lesion cavity was outlined and the area measured by ImageJ, National Institutes of Health, Bethesda, MD, USA. Volume was calculated by multiplying the measured area of each section by the distance between the photographed sections. Single volumes were then summed for a final estimation of total lesion volume.

For quantification of cellular survival, the total number of BrdU-positive cells was calculated. To avoid counting the same cell in adjacent sections, every fifth section was counted (100 μm apart).

To quantify the differentiation pattern of transplanted cells, 100 BrdU cells per animal and specific costaining were analyzed and colocalization with respective cell fate markers in a 50 μm × 50 μm field was counted.

For analysis of anterograde tracing, the section with a clearly recognizable end of the CST was used for the analysis along with four additional adjacent sections. BDA-labeled fibers were quantified at defined distances (0.5 mm, 1 mm, and 2 mm) caudal from the lesion center along the dorso-ventral axis. Labeled fibers are shown as percentage of the total number of fibers at the end of the CST. Only axons fulfilling the criteria of Steward et al. [26] were included in the analysis.

### Statistical analysis

Data are presented as the mean ± standard error of the mean (SEM). The statistical analyzing was carried out with GraphPad Prism 4 (Statcon, Witzenhausen, Germany) and IBM SPSS Statistics 22v software (IBM Corporation, Armonk, NY, USA). Differences in motor behavior were tested using analysis of variance (ANOVA) with repeated measures. The factors were cell groups × time. Because this gave a significant interaction with *p* <0.05, we further tested single days by one-way ANOVA and consecutive post-hoc Tukey’s test (Fig. 4a). Results of the von Frey filament test were analyzed by one-way ANOVA (Fig. 4b).

To test differences in lesion volume, the nonparametric Kruskal–Wallis test followed by Dunn’s post-hoc multiple comparison tests were applied (Fig. 5). When comparing only two groups in immunohistology (BDNF-GFP versus GFP transplants for each differentiation marker or BrdU to assess cell survival), the two-tailed unpaired Student’s *t* test was applied for all markers, except ASPA (one-tailed) (Figs. 6c and 7b). One-way ANOVA for each distance with consecutive post-hoc Tukey’s test was used for anterograde tracing (Fig. 8d). Differences were assumed to be significant if *p* <0.05; nonsignificant differences are not indicated.

## Results

### In vitro differentiation and purification of ESCs

**Fig. 1:**
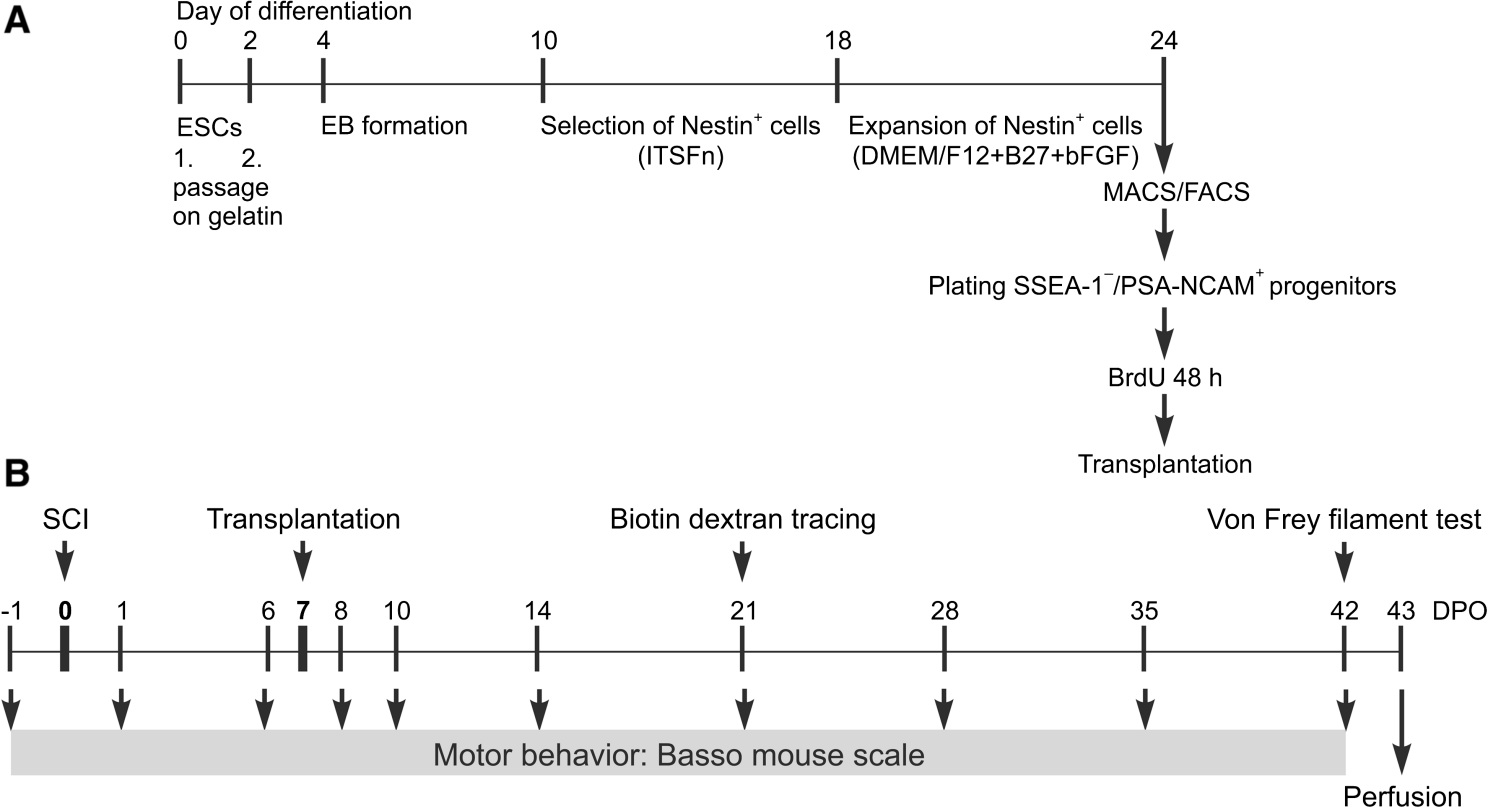
General scheme of ESC differentiation and experimental outline of study. **a** After two passages on gelatin, ESC differentiation started with EB formation, followed by selection of Nestin^+^ cells in ITSFn medium and subsequent expansion in DMEM/F12 + B27 medium in the presence of bFGF. On day 24 of differentiation, cells were purified by FACS and MACS to yield a population of SSEA-1^–^/PSA-NCAM^+^ progenitors, which were labeled with BrdU for 48 hours prior to transplantation. **b** Transplantation was performed on DPO7, the surgery for biotin dextran tracing took place on DPO21, and the von Frey filament test immediately before perfusion on DPO42. Motor behavior by BMS scoring was tested 1 day prior to SCI (DPO–1) and on DPO1, 6, 8, 10, 14, 21, 28, 35, and 42. *bFGF* basic fibroblast growth factor, *BrdU* 5-bromo-2′-deoxyuridine, *DMEM* Dulbecco’s modified Eagle’s medium, *DPO* days post operation, *EB* embryoid body, *ESC* embryonic stem cell, *FACS*, fluorescence-activated cell sorting, *ITSFn* insulin, transferrin, selenium chloride, fibronectin, *MACS* magnetic-activated cell sorting, *PSA-NCAM* polysialylated neural cell adhesion molecule, *SCI* spinal cord injury, *SSEA-1* stage-specific embryonic antigen-1

Mouse ESCs were differentiated into early neural progenitor cells (NPCs), which express PSA-NCAM as a marker. The protocol recapitulates early embryonic development and consists of various differentiation steps, including EB formation, selection, and expansion of Nestin-positive cells (Fig. 1a).

**Fig. 2:**
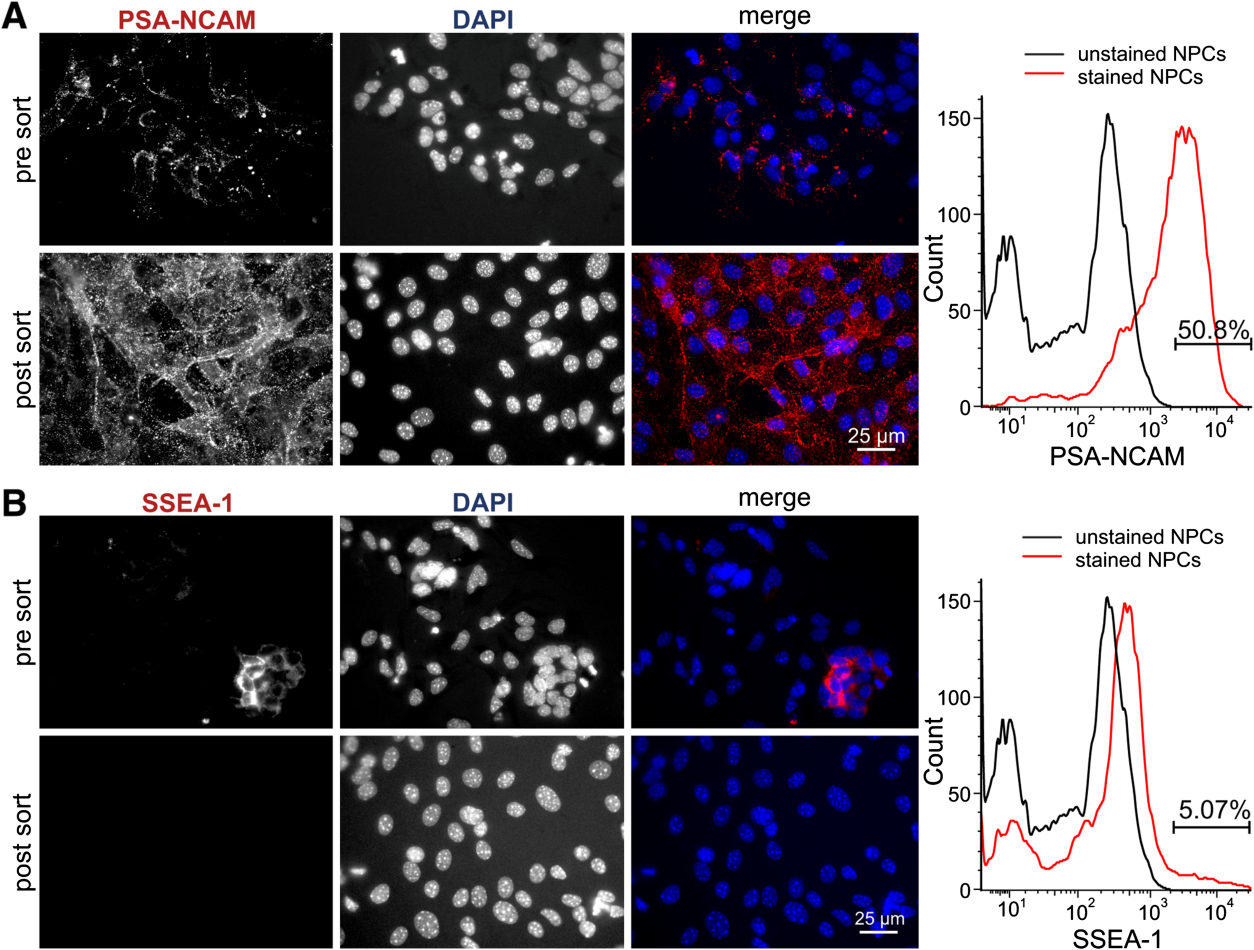
Immunocytochemistry and flow cytometric analysis of NPCs before and after MACS and FACS. Before MACS, 50.8 % of all cells were positive for PSA-NCAM (**a**) (*pre sort*) and 5.07 % positive for SSEA-1 (**b**) (*pre sort*). After MACS and FACS, immunofluorescence showed that cells are positive for PSA-NCAM (**a**) (*post sort*) and negative for SSEA-1 (**b**) (*post sort*). *DAPI* 4′,6-diamidino-2-phenylindol, *NPC* neural progenitor cells, *PSA-NCAM* polysialylated neural cell adhesion molecule, *SSEA-1* stage-specific embryonic antigen-1

**Fig. 3:**
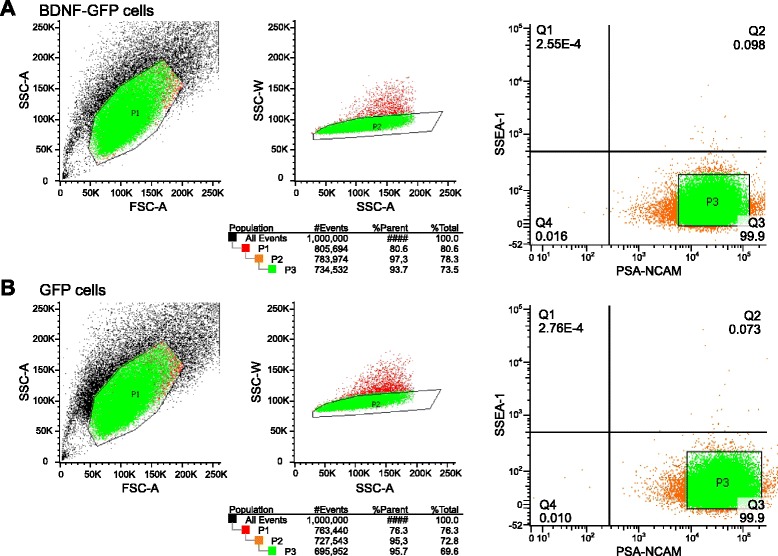
Purification of cells by FACS. BDNF-GFP-expressing cells (**a**) and GFP-expressing control cells (**b**) were sorted by FACS after MACS. The analysis gates were set with reference to negative controls, resulting in a 99.9 % purity of all stained cells (Q3). Sorting gates were set as follows. *Left panels*: the gate (P1) was set for cell size and granularity of the cells. Dead cells and cell debris (shown in *black*) were excluded. *Middle panels*: the gate (P2) was set for single cell sorting. Cell doublets (shown in *red*) were excluded. Right panels show the stringent gate (P3) for the PSA-NCAM sort. *BDNF* brain-derived neurotrophic factor, *FSC* forward scattering, *GFP* green fluorescent protein, *PSA-NCAM* polysialylated neural cell adhesion molecule, *SSC* side scattering, *SSEA-1* stage-specific embryonic antigen-1

On day 24 of differentiation, flow cytometric analysis showed that 50.8 % of all cells were PSA-NCAM-positive (Fig. 2a), and 5.07 % positive for SSEA-1 (Fig. 2b). To obtain a pure neural population for transplantation, the PSA-NCAM-positive fraction was enriched by MACS, and the undifferentiated ESCs positive for SSEA-1 were removed. After MACS, all cells were positive for PSA-NCAM, and no SSEA-1-positive cells were detected (Fig. 2a, b). Cells were analyzed after MACS on the fluorescence-activated cell sorter, revealing that 99.9 % of MACS-sorted cells were positive for PSA-NCAM and negative for SSEA-1 (Fig. 3, quadrant analysis right panels). To deplete single cells positive for SSEA-1, GFP cells and BDNF-GFP cells were subjected to FACS, by applying stringent sorting gates. As shown in Fig. 3a, b, the gating strategy excluded debris and dead cells (obtaining P1) and doublets (P2). The majority of the population was PSA-NCAM^+^/SSEA-1^–^, but single cells were excluded by setting very stringent gates in the population (P3). Double FACS removed remaining SSEA-1-positive cells, which can cause teratoma formation after transplantation. A total of 93.7 % BDNF-GFP cells and 95.7 % GFP cells were sorted from the stained cells, obtaining a pure cell population of PSA-NCAM-positive neural progenitors.

To ensure cell detection in vivo, cells were labeled in vitro with BrdU. Immunocytochemistry revealed that BrdU uniformly labeled all sorted cells (Additional file 1: Figure S1).

### Improved motor function after transplantation of BDNF-GFP neural progenitors

Purified PSA-NCAM^+^ neural progenitors were transplanted directly into the lesion core 7 days after contusion SCI. At different DPO, motor behavior was assessed using the BMS (experimental outline, Fig. 1b).

At DPO1, lesioned animals differed in their BMS scores as they showed variations of their scores ranging from 0.0 to 3.0. The scores on DPO1 showed a significant influence on the scores for the remaining test days DPO6–DPO42 (*p* <0.001 for DPO1 BMS score as a covariate in repeated-measures ANOVA for time (DPO6–DPO42) and “transplantation group” (vehicle, GFP, BDNF-GFP)). Therefore, for the final analyses, the BMS scores for DPO6–DPO42 were normalized to the score on DPO1 and expressed as delta BMS (subtraction of DPO1 score). This resulted in the same delta BMS initial score on DPO1 (delta BMS DPO1 = 0, for all groups).

**Fig. 4:**
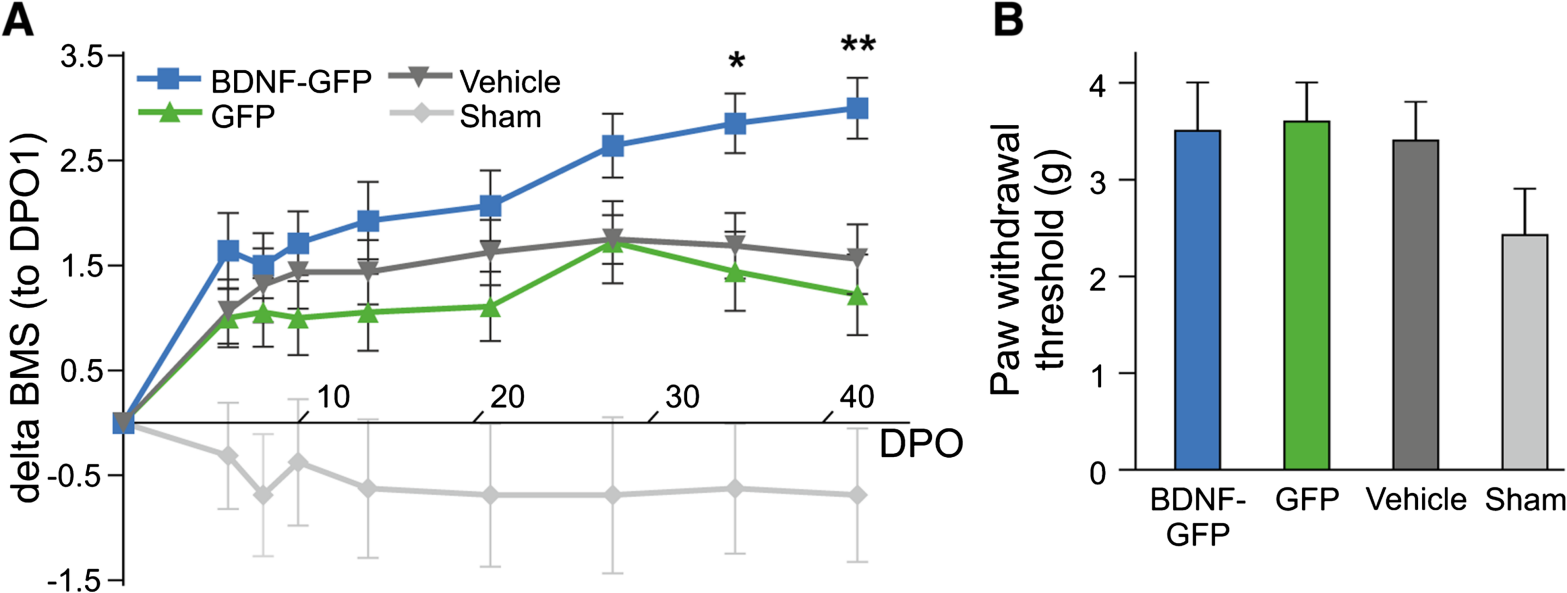
Behavioral assessments. **a** Analysis of delta BMS scores (calculated by subtraction of score from DPO1). The functional recovery of mice with SCI after transplantation of BDNF-GFP cells, GFP cells, or infusion of vehicle (HBSS w/o Ca^2+^/Mg^2+^). The averaged delta BMS scores of the three groups were analyzed via ANOVA with repeated measures (cell groups × time). This gave a significant interaction of cell groups × time of *p* <0.05. Cell group differences were further investigated on single days (DPO35 and DPO42) by one-way ANOVA and consecutive post-hoc Tukey’s test. DPO35, BDNF-GFP to GFP (*p = 0.02); DPO42, BDNF-GFP to GFP (**p = 0.005) and BDNF-GFP to vehicle (*p = 0.026). BDNF-GFP, *n* = 7; GFP, *n* = 9; vehicle, *n* = 8; sham, *n* = 8. **b** Von Frey filament test, representing the average of the stimulus intensity (filament weight in grams) used to determine pain threshold (paw withdrawal threshold) in each group, shown as the average of both hindpaws. BDNF-GFP, *n* = 4; GFP, *n* = 5; vehicle, *n* = 5; sham, *n* = 8. The difference in the number among the animal groups is owing to the viability in the responsiveness of the animals to the stimulus (see Methods for details). BDNF-GFP animals showed no statistically significant change in their mechanical pain threshold as compared with the other groups. The four groups were analyzed by one-way ANOVA. No significant difference among the four groups was detected (*p* = 0.2224). *BDNF* brain-derived neurotrophic factor, *BMS* Basso mouse scale, *DPO* days post operation, *GFP* green fluorescent protein

BDNF-GFP cell transplanted animals displayed a significantly improved motor function compared with GFP cell transplanted or vehicle-treated mice (Fig. 4a). Averaged delta BMS scores on DPO35 showed that BDNF-GFP cell transplanted animals (2.86 ± 0.28) were on average 1.4 points higher scored than GFP cell transplanted animals (1.44 ± 0.38) with *p* = 0.02. On DPO42, this difference became even more evident when BDNF-GFP cell transplanted animals (3.0 ± 0.29) on average revealed a 1.8 points higher score than GFP cell transplanted animals (1.22 ± 0.38) with *p* = 0.005. BDNF-GFP cell transplanted animals also performed significantly better than vehicle-treated mice (1.56 ± 0.33 on DPO42, *p* = 0.026). Unexpectedly, animals transplanted with GFP-expressing NPCs were not significantly different from vehicle-treated mice. Sham group animals displayed negative delta BMS scores, because on DPO1 BMS scores were higher than on the following days tested, which after subtraction of the DPO1 score resulted in negative delta BMS values.

### BDNF-GFP-overexpressing NPCs do not alter pain sensation

As BDNF plays a role in pain modulation, we aimed at proving that BDNF-GFP-overexpressing NPCs are not inducing increased nociception and consequently are not harmful. Therefore, we used the von Frey filament test to assess mechanical allodynia in these mice (Fig. 4b). Pain thresholds were determined in each paw using the up–down method (see Methods). The average of the stimulus intensity (filament weight in grams) that induced the minimal nociceptive response (pain threshold) was calculated for each experimental group and is shown as an average of the right and left paws (Fig. 4b). Results were analyzed by one-way ANOVA. No significant differences among groups were detected (*p* = 0.2224). The tendency of sham animals to respond to lower filament weights than lesioned mice might be explained by the injury itself. Contusion SCI can elicit sensory dysfunction and therefore result in a slightly decreased pain threshold in lesioned mice.

Most importantly, BDNF-GFP cell transplanted animals showed no significant changes in their mechanical pain threshold as compared with GFP cell transplanted mice or the vehicle group. This leads to the conclusion that an increase in BDNF levels, secreted from BDNF-GFP-expressing NPCs in the lesioned spinal cord, does not increase the pain sensation in mice.

### BDNF-GFP NPCs reduce lesion volume in injured mice

**Fig. 5:**
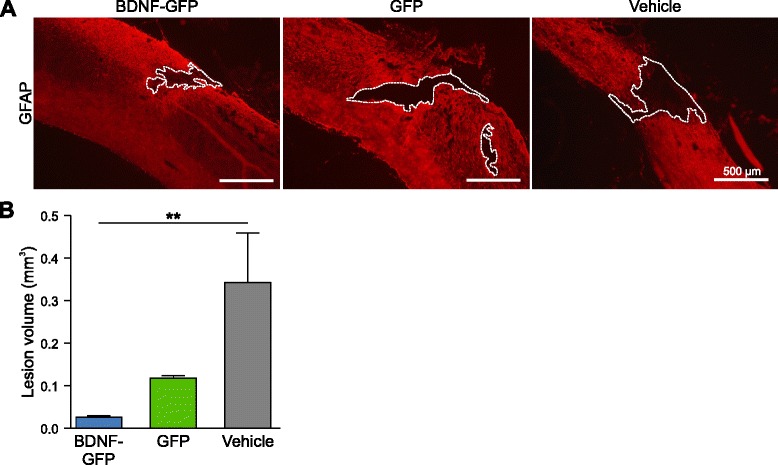
Analysis of lesion volume in injured mice. **a** Representative micrographs of GFAP-stained sections from BDNF-GFP cell-treated, GFP cell-treated, or vehicle-treated spinal cord, showing the center of the lesion. Lesion cavity was determined by negative GFAP staining and outlined (*dotted lines*). **b** Quantitative analysis of lesion volume showed a significant reduction in the BDNF-GFP cell group compared with the vehicle-treated group (*p* = 0.007), but not in the GFP group compared with the vehicle-treated group (*p* = 0.509). The average of *n* = 4 mice ± SEM is presented with **p <0.01, Kruskal–Wallis test with Dunn’s post-hoc test. *BDNF* brain-derived neurotrophic factor, *GFAP* glial fibrillary acidic protein, *GFP* green fluorescent protein

Contusion injury led to a lesion of the spinal cord surrounded by a glial scar. To investigate the potential effect of transplanted NPCs on a histopathological parameter, we assessed the lesion volume by staining for the glial scar marker GFAP (Fig. 5). The lesion area was defined as the area devoid of GFAP staining and outlined on photomicrographs (Fig. 5a). Vehicle-treated animals had an average lesion volume of 0.34 ± 0.11 mm^3^, GFP NPC transplanted animals of 0.12 ± 0.01 mm^3^, and BDNF-GFP NPC transplanted animals of 0.03 ± 0.002 mm^3^. The Kruskall–Wallis test (*p* = 0.01) with Dunn’s post-hoc test revealed a significant reduction in the BDNF-GFP cell group compared with the vehicle-treated group (*p* = 0.007) (Fig. 5b). In contrast, even if showing a trend, GFP NPCs did not lead to a statistical significant reduction in lesion volume (*p* = 0.509).

### Enhanced neuronal and oligodendrocytic in vivo differentiation of BDNF-GFP NPCs

**Fig. 6:**
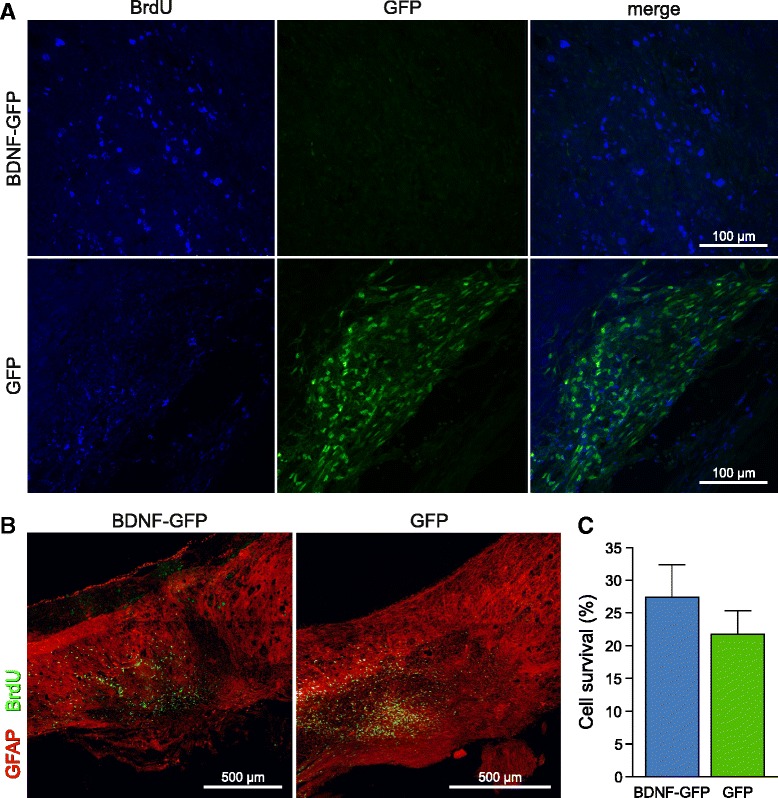
Detection of transplanted NPCs in the spinal cord. **a** Confocal micrographs of grafts of neural progenitors expressing BDNF-GFP (*upper panel*) or GFP (*lower panel*) 5 weeks after cell transplantation in low magnification. GFP cells are positive for BrdU and showed robust GFP expression. However, the GFP signal of BDNF-GFP-expressing cells is very weak and almost undetectable by confocal microscopy, but cells can be efficiently traced by BrdU. **b** Maximum intensity projection of a 20 μm section of a BDNF-GFP cell and GFP cell graft in the spinal cord. BrdU labeled-cells (*green*) are localized near the injury lesion (GFAP negative). **c** Quantification of cell survival in NPC grafted mice: 27.38 ± 4.94 % BDNF-GFP NPCs and 21.74 ± 3.56 % GFP NPCs of 100,000 injected cells were detectable 5 weeks after transplantation. No significant difference was detected among the groups (*p* = 0.4066), unpaired Student’s *t* test. Data represent mean ± SEM. *BDNF* brain-derived neurotrophic factor, *BrdU* 5-bromo-2′-deoxyuridine, *GFAP* glial fibrillary acidic protein, *GFP* green fluorescent protein

Animals were sacrificed by transcardial perfusion on DPO43. To analyze in vivo differentiation of transplanted cells, double immunohistology on sagittal spinal cord sections was performed with cell fate markers and GFP and/or BrdU to trace transplanted cells (Fig. 6). In contrast to GFP-expressing NPCs, transplanted NPCs expressing BDNF-GFP were barely detectable by the GFP signal, even when using an antibody against GFP. This difference in GFP detection might be explainable by the vesicular expression pattern of BDNF-GFP, which we already observed previously [19]. To ensure effective cell tracing also in the BDNF-GFP group, transplanted cells of both groups were labeled in vitro with BrdU right before transplantation (Additional file 1: Figure S1). BrdU staining of transplanted cells in spinal cord sections showed that BDNF-GFP NPCs can efficiently be traced with BrdU 5 weeks after transplantation (Fig. 6a, upper panels).

Both GFP NPCs and BDNF-GFP NPCs integrated into the spinal cord tissue and localized near the lesion site (Fig. 6b). The initial graft contained 100,000 cells per animal; stereological estimation revealed an average of 27.38 ± 4.94 % of BDNF-GFP cells and 21.74 ± 3.56 % of GFP NPCs present 5 weeks following transplantation (Fig. 6c). In general, these values demonstrate proper cellular survival, which does not, however, exclude the possibility of cell proliferation after transplantation. No significant difference was detected between both transplantation groups (*p* = 0.4066). Thus, expression of BDNF-GFP in NPCs did not alter cellular survival after grafting.

**Fig. 7:**
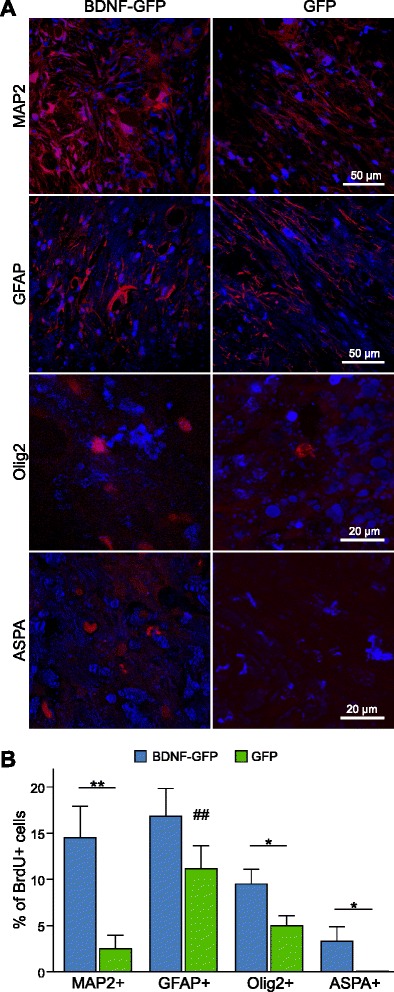
In vivo differentiation of transplanted neural progenitors. **a** Representative confocal micrographs of transplanted BrdU^+^ BDNF-GFP or GFP cells, which are positive for MAP2, GFAP, Olig2, or ASPA. **b** Quantitative analysis of neural progenitor differentiation. Z-stacks were recorded with a confocal laser scanning microscope. Cells positive for BrdU and the respective marker were counted in a 50 μm × 50 μm wide field. BDNF-GFP neural progenitors showed a significant increase towards neuronal (MAP2^+^, *p* = 0.0088) and oligodendrocytic (Olig2^+^, *p* = 0.0418; ASPA^+^, *p* = 0.029) lineage differentiation. Astrocytic differentiation (GFAP^+^) was not significantly changed (*p* = 0.1783) as compared with GFP-expressing neural progenitors. The average of *n* = 6 ± SEM transplanted animals is represented. *p <0.05 and **p <0.01, unpaired Student’s *t* test. Transplants with GFP cells revealed a bias towards astrocytic over neuronal differentiation (^##^ *p* = 0.0067). *ASPA* aspartoacylase, *BDNF* brain-derived neurotrophic factor, *BrdU* 5-bromo-2′-deoxyuridine, *GFAP* glial fibrillary acidic protein, *GFP* green fluorescent protein, *MAP2* microtubule-associated protein 2, *Olig2* oligodendrocyte transcription factor 2

To determine the fate of transplanted neural progenitors, antibodies against markers of all three neural cell lineages were used in double stainings with antibodies against BrdU. Figure 7a shows representative confocal micrographs of grafted BrdU cells, which were positive for the neuronal marker MAP2 or the astrocytic marker GFAP. Furthermore, antibodies against the oligodendrocytic markers Olig2 and ASPA were used. Olig2 is well known for determining oligodendrocyte differentiation, whereas ASPA represents a marker for a later differentiation status. Stained sections were analyzed by the LSM 710, and z-stacks of the lesion core were recorded. To quantify lineage-specific differentiation, the percentage of double-positive cells from all BrdU-positive cells was analyzed. Quantitative assessment of NPC in vivo differentiation in cell transplanted animals showed a tendency for overall increased neural differentiation of BDNF-GFP compared with GFP cells (Fig. 7b). BDNF-GFP neural progenitors differentiated to a greater extent into MAP2-positive neurons (14.5 ± 3.4 %) than GFP NPCs (2.5 ± 1.45 %). Furthermore, differentiation into the oligodendrocytic lineage, assessed by Olig2 staining, was significantly enhanced (9.5 ± 1.6 %) compared with GFP-expressing NPCs (5.0 ± 1.6 %). No ASPA-positive BrdU-labeled cells were found in GFP cell transplanted animals. In contrast, BDNF-GFP cells (3.33 ± 1.54 %) showed a low, but significant proportion in late oligodendrocytic differentiation. Astrocytic differentiation did not significantly differ between both groups. In GFP transplanted grafts, astrocytic differentiation was significantly favored over differentiation into MAP2-positive neurons (*p* = 0.0067), whereas no significant difference between the extent of astrocytic versus neuronal differentiation of BDNF-GFP NPCs was detected (*p* = 0.31).

To test whether transplanted NPCs positive for BrdU but for none of the respective differentiation markers remained in the NPC precursor stage, double staining of BrdU and PSA-NCAM was performed. Double-positive cells were detected in neither cell transplanted group (data not shown), suggesting that NPCs were either differentiating into a neural lineage state, which is not detectable by the used markers, or had undergone transdifferentiation.

### BDNF-GFP transplanted NPCs enhance axonal fiber outgrowth

**Fig. 8:**
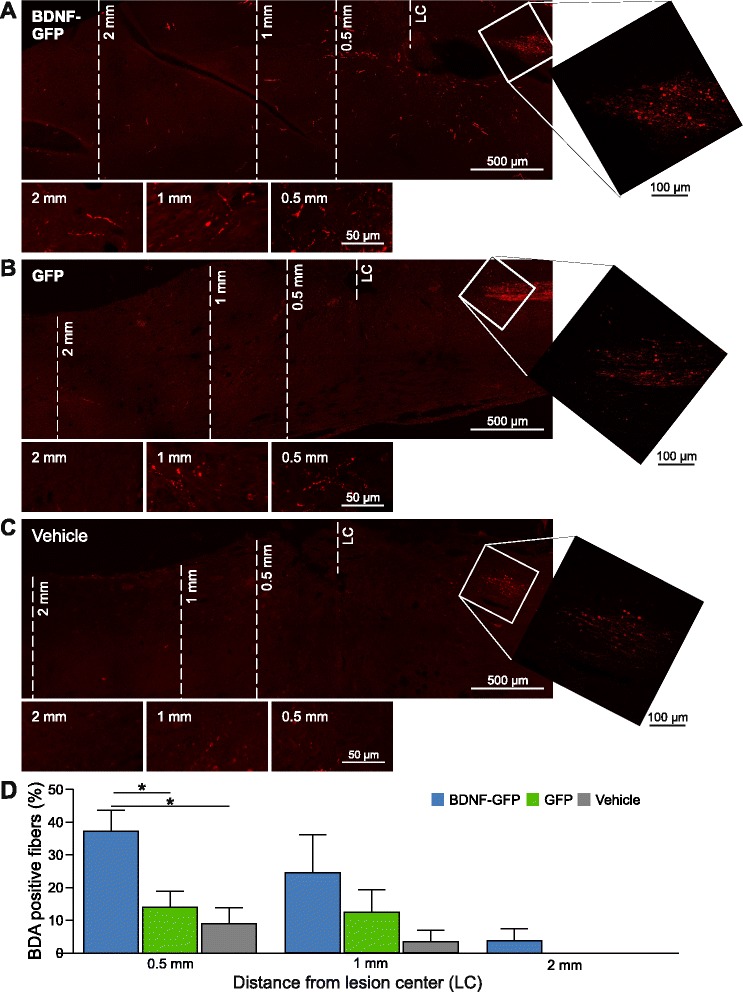
Anterograde tracing of the CST. Overview of confocal micrographs of sagittal sections showing the area of the spinal cord between the CST end and 2 mm caudal to the lesion center (*LC*) of **a** transplanted BDNF-GFP NPCs, **b** transplanted GFP NPCs, and **c** vehicle-treated animals. Pictures below the main panels show details by higher magnification z-stack micrographs of representative areas at 0.5, 1 and 2 mm distance from the LC. *White squares* in the overview images show higher magnification panels of the respective end of the CST. **d** Quantification of BDA-positive CST fibers. The quantity of CST fibers at 0.5 mm distance was significantly elevated in animals transplanted with BDNF-GFP as compared with animals transplanted with GFP NPCs (*p* = 0.035) or compared with vehicle-treated animals (*p* = 0.035). The average of *n* = 4 mice (four adjacent sections per mouse) ± SEM is presented with *p <0.05, one-way ANOVA comparing three groups for each distance with consecutive post-hoc Tukey’s test. *BDA* biotinylated dextran amine, *BDNF* brain-derived neurotrophic factor, *GFP* green fluorescent protein

BDA-traced CST fibers, used as a marker for axonal plasticity, were analyzed in animals which had received either BDNF-GFP cells, GFP NPCs, or vehicle (Fig. 8a–c). Treatment of injured mice with BDNF-GFP-expressing NPCs revealed a significantly higher number (37.25 ± 6.42 %) of BDA-positive fibers than in GFP cell-treated (14.00 ± 4.92 %) or vehicle-treated (9.00 ± 4.85 %) animals 0.5 mm caudal to the lesion site (Fig. 8d). Further distal to the lesion core, at 1 and 2 mm, a similar trend for increased axonal growth induced by BDNF-GFP NPCs was observed. At 2 mm distance caudal to the lesion center, BDA-traced fibers were not detectable anymore in GFP cell-treated or vehicle-treated mice. Furthermore, GFP NPCs did not show any significant improvements of axonal growth compared with vehicle treatment. Altogether, besides the detected effects on stem cell differentiation (see in vivo differentiation of NPCs), BDNF-GFP NPCs were proven to be additionally beneficial by enhancing axonal plasticity in contusion SCI.

## Discussion

In recent years, approaches for treatment of SCI have focused on so-called “combination therapies” which combine modestly effective individual therapies, leading to an enhanced effect when used together [27]. In our approach, we combined the use of predifferentiated ESCs which continuously secrete the neurotrophin BDNF, thus linking cell replacement with neurotrophic supply. BDNF is one of the most studied and promising growth factors for SCI therapy. In rodent animal models, BDNF appears to increase motor functioning shortly after injury [28, 29]. Neural differentiated ESCs transplanted into the injured rodent spinal cord have been shown to differentiate into astrocytes, oligodendrocytes, and neurons, and have been associated with moderate locomotor recovery [3, 5, 6, 30]. Here, we show that ESC-derived neural progenitors, genetically engineered to overexpress BDNF, promote functional recovery in a mouse model of contusion SCI as compared with control cells. In a previous study, we addressed in vitro differentiation of these recombinant ESCs and observed an enhanced neuronal differentiation after retinoic acid treatment of EBs [19]. In order to treat SCI contusion in mice, the protocol was changed to a distinct differentiation protocol obtaining early PSA-NCAM-positive neural progenitors (modified from [21]). These cells were purified by different cell sorting techniques, involving MACS followed by a FACS fine sort to yield a highly pure population of NPCs. Basically, a depletion of remaining tumorigenic SSEA-1-positive undifferentiated ESCs was performed with a subsequent enrichment for PSA-NCAM-positive cells. Consequently, we did not observe any tumor formation or overgrowth after transplantation, which are key concerns after induced pluripotent stem cell (iPSC)/ESC-derived grafting [31, 32].

PSA-NCAM-positive NPCs survived grafting and were detectable 5 weeks after transplantation. As the transplanted early neural precursors were multipotent [20], we could show that they differentiated in vivo into all three neural lineages. In general, BDNF-GFP-overexpressing cells showed an increase in neural differentiation compared with GFP-expressing cells. Particularly, the percentage of MAP2-positive neurons and Olig2 oligodendrocytic precursor cells was significantly higher than in grafts derived from GFP-expressing cells. In addition, no ASPA-positive cells were detected in GFP cell grafts. This was in contrast to BDNF-GFP-overexpressing NPCs, where a small, but significant, proportion of cells differentiated into the late oligodendrocytic stage. The enhanced neuronal and oligodendrocytic differentiation of BDNF-GFP-overexpressing NPCs is of particular importance and might explain, at least in part, the improved motor function in these animals [30]. In general, poor differentiation into neurons and oligodendrocytes was detected when transplanting NPCs. Instead, a strong astrocytic differentiation was observed [33, 34], which might hinder considerable functional recovery, similar to that we observed after transplantation of GFP control cells. Our data provide evidence for BDNF-induced NPC differentiation into both favored lineages in vivo, leading to cell replacement of damaged neurons and oligodendrocytes. We and others have already shown that BDNF enhances neuronal differentiation, as it is known to support differentiation of embryonic, mesenchymal, and NSCs into neurons in vitro and in vivo [8–10, 19]. Furthermore, BDNF is important for oligodendroglial proliferation, differentiation, and myelination [11–13]. Therefore, in terms of cell replacement in SCI, our results demonstrate PSA-NCAM progenitors with a stable secretion of BDNF to be a more potent cellular source than PSA-NCAM (GFP-expressing) progenitors without neurotrophin supply. A remaining question is why transplantation of GFP-expressing NPCs did not enhance functional improvement compared with vehicle treatment, as has been described for other ESC-derived cell transplants into the spinal cord in other investigations [3, 6]. One explanation could be the different directed differentiation and/or the purification of ESCs used in our study. In the aforementioned studies, ESCs were only roughly predifferentiated by EB formation and retinoic acid treatment and were transplanted without further purification. The definite injected cell population then remained unclear. However, it is possible that a mixture of non-neural cells and NSCs/NPCs in different stages of differentiation was advantageous to exert a functional effect [35]. Underlying mechanisms could be either a better differentiation potential of transplanted progenitors or higher cellular survival. In addition, it was reported that overexpression of GFP may generate free radicals, which are toxic to the cell and can lead to apoptosis [36]. However, when we addressed cell survival in our study, we found no significant difference between both transplanted populations and the rate of cell survival appeared normal, when compared with other studies [37, 38]. Nonetheless, lesion size appeared to be differentially affected between both groups. Only BDNF-GFP NPC grafts significantly reduced lesion volume compared with vehicle-treated animals, which underlines the therapeutic effect of BDNF-expressing NPCs based on the behavioral outcome. GFP cells did not ameliorate motor function and only displayed a small, nonsignificant tendency to reduce lesion size.

With our combination therapy approach, we not only aimed for an enhanced/shifted differentiation potential of NPCs overexpressing BDNF, but also for a positive effect on host axonal plasticity by constitutive BDNF secretion. Other studies have demonstrated BDNF-induced axonal regrowth in the presence of a fibroblast or bone marrow stromal cell graft, indicating that BDNF is capable of affecting such regeneration in a suitable environment [17, 39, 40].

Indeed, we were able to show an increase of BDNF-induced axonal plasticity as compared with control cells with our results of CST anterograde axonal tracing. Based on morphological criteria [26, 41], BDA-positive fibers counted caudal to the lesion appeared to present newly formed fibers. However, due to characteristics of contusion injury, we cannot exclude a small percentage of (undetected) sparing. Hence, counted fibers could present a mixture of newly established fibers derived from the site of the lesion and of those sprouting from uninjured fibers, as already described in other studies [42].

To our knowledge, no study has clearly demonstrated growth of corticospinal axons caudal to the lesion site after grafting BDNF-secreting cells. Only in one study was sprouting reported when transplanting BDNF-hypersecreting mesenchymal stem cells, which already had a positive effect on functional outcome [43]. The study of CST growth is particularly important, as this motor projection is critical for human voluntary motor function [41]. So far, only the transduction of fibroblast grafts with neurotrophin-3 led to growth of corticospinal axons and modestly improved functional deficits in SCI [44, 45]. The effect of BDNF on axonal regrowth in other studies [39] could only provide evidence for general axonal regeneration (traced by neurofilament-M staining) and regrowth of rubrospinal neurons [40]. The outcome on motor function was not assessed in either of these studies. In another study where axonal regrowth of spinal motor and sensory neurons was supported, no functional improvement was detected when using transplants of marrow stromal cells overexpressing BDNF [17]. Altogether, we assume that particularly the regrowth of CST axons is important to enhance functional improvement, at least in rodent models of SCI. Why this was evident in our approach and not when transplanting marrow stromal cells overexpressing BDNF [17] could be because we observed a combinatorial effect of ESC-derived precursors and BDNF secretion. It is assumable that neural progenitor grafts create a better neuroprotective environment than marrow stromal cells; for example, by secretion of a cocktail of growth factors and other neurotrophins different from BDNF.

One major concern when transplanting BDNF-overexpressing NPCs into the lesioned spinal cord is a potential increase in neuropathic pain, which would represent a severe side effect of the therapy approach applied here. Indeed, transplantation of NSCs has been shown to improve motor function, but also to induce allodynia in SCI lesioned rats. This effect has been attributed to prevalent astrocytic differentiation and thereby enhanced axonal sprouting, presumably due to secretion of neurotrophins [46]. In general, growth factor delivery has been reported to enhance pain sensitivity in response to non-noxious stimuli in diverse neuropathologies [47–49]. BDNF is one of the neurotrophic factors that have been associated with the development of neuropathic pain [50]. Furthermore, it has been reported that increased BDNF in spinal cord following peripheral nerve damage could result in hyperalgesia in mice [51]. Moreover, BDNF is known to participate in regulating central sensitization and to induce neuropathic pain after SCI [52, 53]. Using the von Frey filament test, we assessed the occurrence of mechanical allodynia in mice after transplanting PSA-NCAM progenitors overexpressing BDNF-GFP or GFP as control. We did not detect any difference in pain threshold between vehicle-treated and both NPC-treated groups, suggesting that neither the transplantation of NPCs nor the overexpression of BDNF led to alterations in nociception in these mice. The discrepancy between our study and the data reported by Hofstetter et al. [46] may be caused by the fact that we used further differentiated neural cells, and that PSA-NCAM-positive progenitors are less harmful than naïve NSCs and thus have less impact on affecting pain sensitivity in mice. Our BDNF overexpression, which leads to approximately eight times higher levels than the endogenous BDNF in ESC-derived differentiated neurons [19], is therefore a moderate overexpression, which does not appear to induce the development of allodynia.

## Conclusions

The use of BDNF-overexpressing PSA-NCAM-positive NSCs is a conceivable therapeutic approach for the treatment of incomplete SCI. BDNF promoted NPC differentiation into favored neuronal and oligodendrocytic cells and promoted host axonal plasticity. Both effects resulted in an improved motor recovery without inducing allodynia or tumor formation.

In future, the validity of our combinatorial therapeutic approach could be evaluated with iPSCs overexpressing BDNF, which recently have been produced from rats and used to modulate stress-related pathology [54]. However, those cells have not been transduced by targeted BDNF gene transfer using homologous recombination as we used [19]. Gene targeting is a much safer approach, avoiding unwanted random integration, which might affect genome function.

## Additional file

Additional file 1: Figure S1. Efficient BrdU labeling and in vitro detection of sorted neural progenitors. **A** Representative bright-field (*BF*) image showing sorted PSA-NCAM-positive cells. **B** BrdU treatment resulted in a uniform fluorescent staining of the nuclei (*blue*). **C** All cells were positively labeled with BrdU (merge image). (PDF 3019 kb)

## Abbreviations

ANOVA: Analysis of variance
APC: Allophycocyanin
ASPA: Aspartoacylase
BDA: Biotinylated dextran amine
BDNF: Brain-derived neurotrophic factor
bFGF: Basic fibroblast growth factor
BMS: Basso mouse scale
BrdU: 5-Bromo-2′-deoxyuridine
CST: Corticospinal tract
DAPI: 4′,6-Diamidino-2-phenylindol
DMEM: Dulbecco’s modified Eagle’s medium
DPO: Days post operation
EB: Embryoid body
ESC: Embryonic stem cell
FACS: Fluorescence-activated cell sorting
GFAP: Glial fibrillary acidic protein
GFP: Green fluorescent protein
HBSS w/o Ca^2+/^Mg^2+^: Hank’s Balanced Salt Solution without calcium and magnesium
iPSC: Induced pluripotent stem cell
ITSFn: Insulin, transferrin, selenium chloride, and fibronectin
LIF: Leukemia inhibitory factor
MACS: Magnetic-activated cell sorting
MAP2: Microtubule-associated protein 2
NPC: Neural progenitor cell
NSC: Neuronal stem cell
Olig2: Oligodendrocyte transcription factor 2
PBS: Phosphate-buffered saline
PBS-TX: Phosphate-buffered saline with Triton X-100
PE: r-Phycoerythrin
PSA-NCAM: Polysialylated neural cell adhesion molecule
SCI: Spinal cord injury
SEM: Standard error of the mean
SSEA-1: Stage-specific embryonic antigen-1

## References

[1] Thuret S, Moon LD, Gage FH (2006). Therapeutic interventions after spinal cord injury. Nat Rev Neurosci..

[2] Li J, Lepski G. Cell transplantation for spinal cord injury: a systematic review. Biomed Res Int. 2013. doi:10.1155/2013/786475. 10.1155/2013/786475 PMC3581246 23484157

[3] McDonald JW, Liu XZ, Qu Y, Li S, Mickey SK, Turetsky D (1999). Transplanted embryonic stem cells survive, differentiate and promote recovery in injured rat spinal cord. Nat Med..

[4] Cummings BJ, Uchida N, Tamaki SJ, Salazar DL, Hooshmand M, Summers R (2005). Human neural stem cells differentiate and promote locomotor recovery in spinal cord-injured mice. Proc Natl Acad Sci U S A..

[5] Keirstead HS, Nistor G, Bernal G, Totoiu M, Cloutier F, Sharp K (2005). Human embryonic stem cell-derived oligodendrocyte progenitor cell transplants remyelinate and restore locomotion after spinal cord injury. J Neurosci..

[6] Marques SA, Almeida FM, Fernandes AM, dos Santos SC, Cadilhe DV, Rehen SK (2010). Predifferentiated embryonic stem cells promote functional recovery after spinal cord compressive injury. Brain Res..

[7] Huang EJ, Reichardt LF (2001). Neurotrophins: roles in neuronal development and function. Annu Rev Neurosci..

[8] Kang SK, Lee RH, Jung JS (2001). Effect of brain-derived neurotrophic factor on neural differentiation of mouse embryonic stem cells and neural precursor cells. Neurosci Res Commun..

[9] Ortega JA, Alcántara S (2010). BDNF/Mapk/Erk-induced BMP7 expression in the developing cerebral cortex induces premature radial glia differentiation and impairs neuronal migration. Cereb Cortex..

[10] Trzaska KA, Rameshwar P (2011). Dopaminergic neuronal differentiation protocol for human mesenchymal stem cells. Methods Mol Biol..

[11] Du Y, Fischer TZ, Clinton-Luke P, Lercher LD, Dreyfus CF (2006). Distinct effects of p75 in mediating actions of neurotrophins on basal forebrain oligodendrocytes. Mol Cell Neurosci..

[12] Van’t Veer A, Du Y, Fischer TZ, Boetig DR, Wood MR, Dreyfus CF (2009). Brain-derived neurotrophic factor effects on oligodendrocyte progenitors of the basal forebrain are mediated through Trkb and the map kinase pathway. J Neurosci Res..

[13] Vondran MW, Clinton-Luke P, Honeywell JZ, Dreyfus CF (2010). BDNF+/− mice exhibit deficits in oligodendrocyte lineage cells of the basal forebrain. Glia..

[14] Liu Y, Kim D, Himes BT, Chow SY, Schallert T, Murray M (1999). Transplants of fibroblasts genetically modified to express BDNF promote regeneration of adult rat rubrospinal axons and recovery of forelimb function. J Neurosci..

[15] Jin Y, Tessler A, Fischer I, Houle JD (2000). Fibroblasts genetically modified to produce BDNF support regrowth of chronically injured serotonergic axons. Neurorehabil Neural Repair..

[16] Jin Y, Fischer I, Tessler A, Houle JD (2002). Transplants of fibroblasts genetically modified to express BDNF promote axonal regeneration from supraspinal neurons following chronic spinal cord injury. Exp Neurol..

[17] Lu P, Jones LL, Tuszynski MH (2005). BDNF-expressing marrow stromal cells support extensive axonal growth at sites of spinal cord injury. Exp Neurol..

[18] Bonner JF, Blesch A, Neuhuber B, Fischer I (2010). Promoting directional axon growth from neural progenitors grafted into the injured spinal cord. J Neurosci Res..

[19] Leschik J, Eckenstaler R, Nieweg K, Lichtenecker P, Brigadski T, Gottmann K (2013). Embryonic stem cells stably expressing BDNF-GFP exhibit a BDNF-release-dependent enhancement of neuronal differentiation. J Cell Sci..

[20] Kim D-S, Lee DR, Kim H-S, Yoo J-E, Jung SJ, Lim BY (2012). Highly pure and expandable PSA-NCAM-positive neural precursors from human ESC and iPSC-derived neural rosettes. PLoS One..

[21] Bernreuther C, Dihne M, Johann V, Schiefer J, Cui Y, Hargus G (2006). Neural cell adhesion molecule L1-transfected embryonic stem cells promote functional recovery after excitotoxic lesion of the mouse striatum. J Neurosci..

[22] Barral S, Ecklebe J, Tomiuk S, Tiveron MC, Desoeuvre A, Eckardt D (2013). Efficient neuronal in vitro and in vivo differentiation after immunomagnetic purification of mESC derived neuronal precursors. Stem Cell Res..

[23] Behringer R, Gertsenstein M, Vintersten Nagy K, Nagy A (2014). Introduction of foreign DNA into embryonic stem cells. Manipulating the mouse embryo. A laboratory manual.

[24] Basso DM, Fisher LC, Anderson AJ, Jakeman LB, McTigue DM, Popovich PG (2006). Basso mouse scale for locomotion detects differences in recovery after spinal cord injury in five common mouse strains. J Neurotrauma..

[25] Chaplan SR, Bach FW, Pogrel JW, Chung JM, Yaksh TL (1994). Quantitative assessment of tactile allodynia in the rat paw. J Neurosci Methods..

[26] Steward O, Zheng B, Tessier-Lavigne M (2003). False resurrections: distinguishing regenerated from spared axons in the injured central nervous system. J Comp Neurol..

[27] Rosner J, Avalos P, Acosta F, Liu J, Drazin D. The potential for cellular therapy combined with growth factors in spinal cord injury. Stem Cells Int. 2012. doi:10.1155/2012/826754. 10.1155/2012/826754 PMC3471462 23091499

[28] Houweling DA, van Asseldonk JTH, Lankhorst AJ, Hamers FPT, Martin D, Bär PR (1998). Local application of collagen containing brain-derived neurotrophic factor decreases the loss of function after spinal cord injury in the adult rat. Neurosci Lett..

[29] Namiki J, Kojima A, Tator CH (2000). Effect of brain-derived neurotrophic factor, nerve growth factor, and neurotrophin-3 on functional recovery and regeneration after spinal cord injury in adult rats. J Neurotrauma..

[30] Kumagai G, Okada Y, Yamane J, Nagoshi N, Kitamura K, Mukaino M (2009). Roles of ES cell-derived gliogenic neural stem/progenitor cells in functional recovery after spinal cord injury. PLoS One..

[31] Nori S, Okada Y, Yasuda A, Tsuji O, Takahashi Y, Kobayashi Y (2011). Grafted human-induced pluripotent stem-cell-derived neurospheres promote motor functional recovery after spinal cord injury in mice. Proc Natl Acad Sci U S A..

[32] Tsuji O, Miura K, Okada Y, Fujiyoshi K, Mukaino M, Nagoshi N (2010). Therapeutic potential of appropriately evaluated safe-induced pluripotent stem cells for spinal cord injury. Proc Natl Acad Sci U S A..

[33] Ogawa Y, Sawamoto K, Miyata T, Miyao S, Watanabe M, Nakamura M (2002). Transplantation of in vitro-expanded fetal neural progenitor cells results in neurogenesis and functional recovery after spinal cord contusion injury in adult rats. J Neurosci Res..

[34] Vroemen M, Aigner L, Winkler J, Weidner N (2003). Adult neural progenitor cell grafts survive after acute spinal cord injury and integrate along axonal pathways. Eur J Neurosci..

[35] Pearse DD, Sanchez AR, Pereira FC, Andrade CM, Puzis R, Pressman Y (2007). Transplantation of Schwann cells and/or olfactory ensheathing glia into the contused spinal cord: survival, migration, axon association, and functional recovery. Glia..

[36] H-Shg L, Jan M-S, Chou C-K, Chen P-H, Ke N-J (1999). Is green fluorescent protein toxic to the living cells?. Biochem Biophys Res..

[37] Salewski RP, Mitchell RA, Shen C, Fehlings MG (2015). Transplantation of neural stem cells clonally derived from embryonic stem cells promotes functional recovery after murine spinal cord injury. Stem Cells Dev..

[38] Karimi-Abdolrezaee S, Eftekharpour E, Wang J, Morshead CM, Fehlings MG (2006). Delayed transplantation of adult neural precursor cells promotes remyelination and functional neurological recovery after spinal cord injury. J Neurosci..

[39] McTigue DM, Horner PJ, Stokes BT, Gage FH (1998). Neurotrophin-3 and brain-derived neurotrophic factor induce oligodendrocyte proliferation and myelination of regenerating axons in the contused adult rat spinal cord. J Neurosci..

[40] Kwon BK, Liu J, Messerer C, Kobayashi NR, McGraw J, Oschipok L (2002). Survival and regeneration of rubrospinal neurons 1 year after spinal cord injury. Proc Natl Acad Sci U S A..

[41] Tuszynski MH, Steward O (2012). Concepts and methods for the study of axonal regeneration in the CNS. Neuron..

[42] Boato F, Rosenberger K, Nelissen S, Geboes L, Peters EM, Nitsch R (2013). Absence of Il-1β positively affects neurological outcome, lesion development and axonal plasticity after spinal cord injury. J Neuroinflammation..

[43] Sasaki M, Radtke C, Tan AM, Zhao P, Hamada H, Houkin K (2009). BDNF-hypersecreting human mesenchymal stem cells promote functional recovery, axonal sprouting, and protection of corticospinal neurons after spinal cord injury. J Neurosci..

[44] Grill R, Murai K, Blesch A, Gage FH, Tuszynski MH (1997). Cellular delivery of neurotrophin-3 promotes corticospinal axonal growth and partial functional recovery after spinal cord injury. J Neurosci..

[45] Tuszynski MH, Grill R, Jones LL, Brant A, Blesch A, Löw K (2003). Nt-3 gene delivery elicits growth of chronically injured corticospinal axons and modestly improves functional deficits after chronic scar resection. Exp Neurol..

[46] Hofstetter CP, Holmstrom NAV, Lilja JA, Schweinhardt P, Hao J, Spenger C (2005). Allodynia limits the usefulness of intraspinal neural stem cell grafts; directed differentiation improves outcome. Nat Neurosci..

[47] Eriksdotter Jönhagen M, Nordberg A, Amberla K, Bäckman L, Ebendal T, Meyerson B (1998). Intracerebroventricular infusion of nerve growth factor in three patients with alzheimer’s disease. Dement Geriatr Cogn Disord..

[48] Hao J-X, Ebendal T, Xu X-J, Wiesenfeld-Hallin Z, Eriksdotter JM (2000). Intracerebroventricular infusion of nerve growth factor induces pain-like response in rats. Neurosci Lett..

[49] Jubran M, Widenfalk J (2003). Repair of peripheral nerve transections with fibrin sealant containing neurotrophic factors. Exp Neurol..

[50] Constandil L, Aguilera R, Goich M, Hernández A, Alvarez P, Infante C (2011). Involvement of spinal cord BDNF in the generation and maintenance of chronic neuropathic pain in rats. Brain Res Bull..

[51] Yajima Y, Narita M, Usui A, Kaneko C, Miyatake M, Narita M (2005). Direct evidence for the involvement of brain-derived neurotrophic factor in the development of a neuropathic pain-like state in mice. J Neurochem..

[52] Biggs JE, Lu VB, Stebbing MJ, Balasubramanyan S, Smith PA (2010). Is BDNF sufficient for information transfer between microglia and dorsal horn neurons during the onset of central sensitization?. Mol Pain..

[53] Wu J, Renn CL, Faden AI, Dorsey SG (2013). Trkb.T1 contributes to neuropathic pain after spinal cord injury through regulation of cell cycle pathways. J Neurosci.

[54] Liu G, Rustom N, Litteljohn D, Bobyn J, Rudyk C, Anisman H (2014). Use of induced pluripotent stem cell derived neurons engineered to express BDNF for modulation of stressor related pathology. Front Cell Neurosci..

