# Supplementary figures and images for: PSA-NCAM positive neural progenitors stably expressing BDNF promote functional recovery in a mouse model of spinal cord injury
Source: Stem Cell Res Ther. 2016 Jan 13;7:11. doi: 10.1186/s13287-015-0268-x (PMC4712602; doi:10.1186/s13287-015-0268-x)

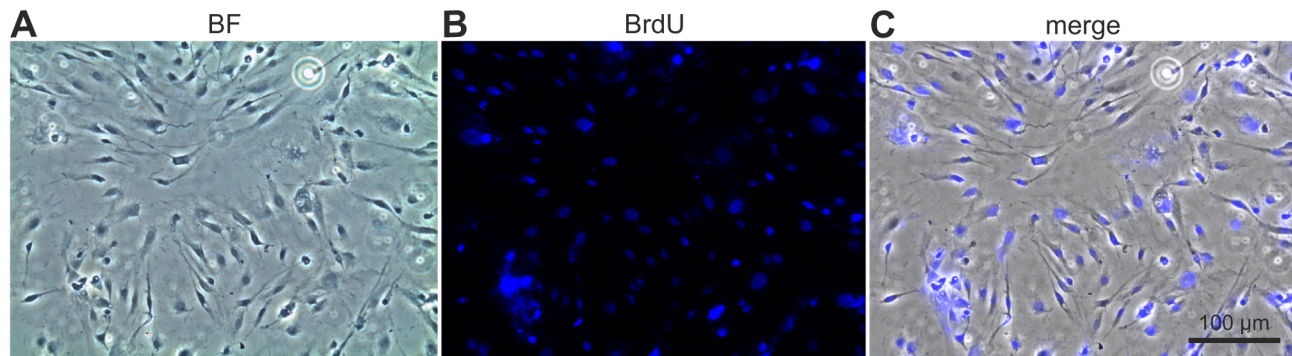

**Figure S1**

Supplement: Additional file 1: Figure S1. — Efficient BrdU labeling and in vitro detection of sorted neural progenitors. A Representative bright-field (BF) image showing sorted PSA-NCAM-positive cells. B BrdU treatment resulted in a uniform fluorescent staining of the nuclei (blue). C All cells were positively labeled with BrdU (merge image). (PDF 3019 kb) [file 13287_2015_268_MOESM1_ESM.pdf]
